# hZIP1 Inhibits Progression of Clear Cell Renal Cell Carcinoma by Suppressing NF-kB/HIF-1α Pathway

**DOI:** 10.3389/fonc.2021.759818

**Published:** 2021-12-02

**Authors:** Bo Zhan, Xiao Dong, Yulin Yuan, Zheng Gong, Bohan Li

**Affiliations:** Department of Urology, The First Hospital of China Medical University, Shenyang, China

**Keywords:** anaerobic glycolysis, ccRCC, HIF-1α, hZIP1, NF-kB pathway

## Abstract

**Purpose:**

Accumulating literature has suggested that hZIP1 and HIF-1α play vital roles in the tumor process of clear cell renal cell carcinoma (ccRCC). However, the functional roles of hZIP1 and HIF-1α in ccRCC remain largely unknown.

**Methods:**

HIF-1α protein level was evaluated by a western blot in ccRCC tissues and cell lines. ccRCC cell lines were transfected with HIF-1α-siRNA to downregulate the expression level of HIF-1α. Then the proliferative, migratory and invasive abilities of ccRCC cells *in vitro* were detected by real-time cell analysis (RTCA) assay, wound healing assay and transwell assay, respectively. The role of HIF-1α *in vivo* was explored by tumor implantation in nude mice. Then the effect on glycolysis‐related proteins was performed by western blot after hZIP1 knockdown (overexpression) or HIF-1α knockdown. The effect on NF‐kB pathway was detected after hZIP1 overexpression.

**Results:**

HIF-1α was markedly downregulated in ccRCC tissues compared with normal areas. But HIF-1α presented almost no expression in HK-2 and ACHN cells. Immunofluorescence indicated HIF-1α and PDK1 expression in both the cytoplasm and nucleus in ccRCC cells. Downregulation of HIF-1α suppressed ccRCC cell proliferation, migration, and invasion and resulted in smaller implanted tumors in nude mice. Furthermore, hZIP1 knockdown elevated HIF-1α protein levels and PDK1 protein levels in ccRCC cells. Interestingly, a sharp downregulated expression of HIF-1α was observed after hZIP1 overexpression in OSRC-2 and 786-O cells, which resulted from a downtrend of NF-*k*B1 moving into the cell nucleus.

**Conclusion:**

Our work has vital implications that hZIP1 suppresses ccRCC progression by inhibiting NF-kB/HIF-1α pathway.

## Introduction

Clear cell renal cell carcinoma (ccRCC) is one of the most common malignant tumors in the urinary system, accounting for approximately 2–3% of malignant tumors in adults, and its incidence has been increasing steadily in our country. A certain number of people cannot avoid tumor recurrence or metastasis after surgical resection, where Fuhrman IV has poor prognosis, and the five-year survival rate is 20% (1, 2). Metastatic renal cancer is not sensitive to either radiotherapy or chemotherapy, and immunotherapy also has limited effects (3). Therefore, studying the mechanism and molecular targets has important implications for the early diagnosis and treatment of ccRCC.

The evolution of ccRCC is accompanied by changes in cell energy metabolism; that is, from aerobic phosphorylation to aerobic glycolysis (the Warburg effect) (4, 5). Hypoxia-inducible factor (HIF) is a vital transcription factor and regulator of cell responses to hypoxic conditions and can be divided into HIF-1 and HIF-2. Hypoxia-inducible factor 1 (HIF-1) is a highly conserved member of the basic helix-loop-helix (bHLH) transcription factor family, which is composed of α and β subunit dimers. After the α and β subunits dimerize, relevant downstream genes such as glycolysis-related enzyme genes start to work, so HIF-1α and HIF-2α are probably key molecules for glycolysis and cell energy metabolism (6, 7). Furthermore, HIF-1α can be activated by a number of cancer cell signaling pathways and plays a crucial role in the carcinogenesis process (8, 9). In the study on the pathogenesis of ccRCC, the most prominent genes were all related to the expression of HIF-1α. For example, VHL (Von Hippel–Lindau) is inactivated by mutation or transcriptional regulation in ccRCC, and HIF-1α becomes unable to be ubiquitinated. Moreover, some downstream proteins such as VEGF are elevated to promote tumorigenesis progression (10).

At the same time, the relationship between microelements and tumor progression has started to become viable (11–13). Among them, a regulatory protein of Zn^2+^, hZIP1 (SLC39A1, NCBI Reference Sequence: NM_001271960.2), has demonstrated significant roles in both cellular metabolism and tumorigenesis (12–14). A number of studies have proven significant correlations of hZIP1 low expression with ovarian, colon, gastric, and prostate tumors (15, 16). Above all, the decreased expression of hZIP1 in prostate tumor tissues can cause metabolic alterations in epithelial cells of the prostate by facilitating citric acid transport out of the mitochondria (15). It is speculated that hZIP1 participates in the metabolic progression of Zn^2+^ and plays a vital role in the development of tumors. Moreover, the enhancement of HIF-1α-related glucose metabolism in tumor progression may be influenced by the expression reduction or fault of hZIP1. Our previous work showed that hZIP1 demonstrated lower expression in ccRCC tissues than in normal tissues, and after knockdown of hZIP1, the proliferation and invasion of renal cancer cells increased (17). This outcome illustrated that hZIP1 acted as a tumor suppressor in ccRCC. In this study, we aimed to clarify the relationship between hZIP1, HIF-1α, and cancer cell energy metabolism. To this end, we detected the expression and localization of HIF-1α protein in renal normal/ccRCC tissues and renal cancer cells. Then, we explored the biological function and probable mechanism of HIF-1α and hZIP1 by controlling their expression.

## Materials and Methods

### Bioinformatics Analyses

To better comprehend the possible relationships between patient survival status or HIF-1α mRNA expression in ccRCC tissues compared to normal renal tissues, TCGA platform analysis is available at the following links: http://gepia.cancer-pku.cn/detail.php and http://ualcan.path.uab.edu/index.html. The immunohistochemistry (IHC) images of HIF-1α expression in normal renal tissues and ccRCC tissues are presented in The Human Protein Atlas database, see the following link: https://www.proteinatlas.org/.

### Cell Culture

ccRCC cells were routinely cultured in RPMI-1640 medium (786-O and OSRC-2 cells), MEM medium (ACHN cells), and McCoy’s 5A medium (Caki-1 cells) supplemented with 10% fetal bovine serum (FBS) (TBD, Tianjin, China). HK-2 cells (immortalized renal tubular epithelial cells) was cultured in F12 medium supplemented with 10% FBS. All mediums were obtained from HyClone Inc. All cells were purchased from the Chinese Academy of Sciences Cell Bank. All cells were incubated in a humidified atmosphere at 37°C under normoxia (20% O_2_ and 5% CO_2_).

### siRNA and Plasmid (Lentivirus) Transfection

Small interfering RNA (siRNA) was purchased from GenePharma (Shanghai, China). Caki-1 and OSRC-2 cells were transfected with siRNA (HIF-1α-siRNA, hZIP1-siRNA, and NC-siRNA). OSRC-2 cells were transfected with plasmids (HIF-1α-shRNA and NC-shRNA) for the animal experiment. The transfection experiment were performed in 6-well plates using Lipofectamine 3000 (Invitrogen, Carlsbad, CA) in accordance with the manufacturer’s instructions. Lentiviral transfection was also strictly following the specifications. After 48 h, the siRNA-transfected renal cancer cells were harvested for subsequent studies; plasmid (lentivirus)-transfected renal cancer cells were continually cultivated with puromycin (2 μg/ml) to select positive clones. The siRNA sequences were: HIF-1α-siRNA-1: 5’-GCCGCUCAAUUUAUGAAUATT-3’,5’-UAUUCAUAAAUUGAGCGGCTT-3’. HIF-1α-siRNA-2: 5’-CCACCACUGAUGAAUUAAATT-3’, 5’-UUUAAUUCAUCAGUGGUGGTT-3’. hZIP1-siRNA-1: 5’-GCAUGACACCUCUAGGCAUTT-3’, 5’-AUGCCUAGAGGUGUCAUGCTT-3’, hZIP1-siRNA-2: 5’-GCUGUUGCAGAGCCACCUUTT-3’, 5’-AAGGUGGCUCUGCAACAGCTT-3’. HIF-1α-shRNA target sequence: cgGCGAAGTAAAGAATCTGAA. Lentiviral vector transcript: NM_014437. The OSRC-2 cells overexpressed hZIP1 were indicated as OSRC-2-OEhZIP1, and the control cells were OSRC-2-OENC. The 786-O cells overexpressed hZIP1 were indicated as 786-O-OEhZIP1, and the control cells were 786-O-OENC.

### Nude Mice Model of Tumor Implantation

Animal experiments were approved by the Ethics Committee of China Medical University. There is no obvious difference of hZIP1 and HIF-1α expression between different genders. For the convenience of feeding, we used female mice for the animal experiment. OSRC-2 cells transfected with HIF-1α-shRNA and NC-shRNA plasmid for nude mouse experiments were injected at 1 × 10^6^ cells/mouse into the axilla of 4-week-old female SPF nude mice (from Vitalriver, China) (n = 5 mice/group). After approximately 4 weeks, all mice were killed, and tumors were resected and weighed. The animal experiments were approved by the Institutional Research Ethical Committee of China Medical University.

### Patients and Specimens

The ccRCC tissues were randomly selected from 32 patients (20 males and 12 females). Radical resection was performed on all patients in the First Affiliated Hospital of China Medical University between 2018 and 2020. All cases were diagnosed with ccRCC by pathological methods. This study was approved by the Ethics Committee of China Medical University and all patients had provided the informed consent. The clinicopathological information of ccRCC patients is presented in **Figure S1**.

### Western Blotting Analysis

The total protein was isolated using RIPA (radioimmuno-precipitation assay) buffer and the protease inhibitor phenylmethanesulfonyl fluoride (PMSF). Nuclear proteins were extracted according to the Nuclear Protein Extraction Kit (Beyotime Biotechnology). Lamin B1 (a loading control, 1:1,000) was purchased from Cell Signaling Technology. Fifty micrograms of standardized proteins were separated using SDS-PAGE (10%) and transferred onto PVDF (polyvinylidene fluoride) membranes (Bio-Rad, USA). After the transferred membranes were blocked in milk at 37°C for an hour on a shaking table, they were incubated overnight with primary antibodies [HIF-1α (1:1,000), NF-κB1 (1:1,000), NF-κB-p65 (1:1,000) (Santa Cruz Biotechnology), hZIP1 (1:500), β-actin (1:5,000), GLUT1 (1:1,000), LDHA (1:1,000), PDK1 (1:2,000) (Cell Signaling Technology)] at 4°C. The following day, membranes were washed using TBS-T (Tris-buffered saline mixed with Tween 20) and then incubated with TBST-conjugated secondary antibodies (1:5,000) for 1 h at 37°C on a shaking table. The EC3 Imaging System (UVP Inc., Cambridge, UK) was used to determine the expression of target proteins.

### Immunofluorescence Staining

Renal cancer cells 786-O, Caki-1, and OSRC-2 (5,000 cells per well) were incubated overnight with antibodies against HIF-1α (1:50) (Santa Cruz Biotechnology) and PDK1 (1:50) (Cell Signaling Technology) in a 24-well plate (Corning, NY, USA). Then, Alexa Fluor 488-conjugated secondary antibody was added to these cells for 1 h (in the dark at 37°C) followed by DAPI stained on the nucleus. Finally, an inverted fluorescence microscopy (Olympus, Tokyo, Japan) was used for images.

### Real Time Cell Analysis (RTCA) Assay

RTCA (ACEA Biosciences, USA) was used to monitor cell viability according to the manufacturer’s instructions. An xCELLigence System (Roche Applied Sciences) was used for recording the cell growth curves automatically. The cell index was used to observe cell status (such as cell numbers and cell attachment). When cells adhered to the surface of the plate and then influenced, the software recorded electrical values and transformed them into a cell index. First, the background value was measured after the culture medium (50 μl) was added to the plate. Then, Caki-1 or OSRC-2 cells (100 μl, 3,000 cells per well) (transfected with NC-siRNA, HIF-1α-siRNA-1 or HIF-1α-siRNA-2, respectively) were seeded into plates. The data were documented with ACEA Biosciences RTCA software 2.0 and analyzed by GraphPad Prism 7.0.

### Wound Healing Assay

ccRCC cells (HIF-1α-siRNA was performed for 24 h, 1 ∗ 10^4^ cells per well) were inoculated into a 24-well plate with medium containing 10% FBS. Then, a 1 ml plastic tip was used to make a scratch when the cells had conjugated to 90%. The plate was washed two times with PBS to remove cellular fragments and then replaced with a serum-free medium for 24 h. Wound closure was captured by an inverted microscopy and ImageJ was used for calculating the wound areas. All results were repeated for three times.

### Transwell Invasion Assay

In the invasion assay, the upper chambers (8.0 μm Invasion Chambers, Coring) were pretreated with Matrigel (BD Biosciences, Franklin Lakes, USA) (Matrigel: serum-free medium was 1:5, 25 μl per chamber). ccRCC cells (1 × 10^5^) were mixed with 200 μl medium (2% FBS) and then seeded in the upper chamber. The lower chamber was filled with 600 μl of the same medium (10% FBS) as the upper chamber. After incubation for 48 h, PBS was used to wash the upper chamber twice, and then a cotton swab was used to remove the non-invaded cells in the upper chamber. The invaded cells were stained with crystal violet for 15 min, and then a microscope (Olympus, Tokyo, Japan) was used to determine the number of invaded cells (randomly counted five fields of cells). All results were repeated for three times.

### Statistical Analysis

Data are represented as the mean ± standard deviation (SD). Paired Student’s t-test was used for examining the expression of HIF-1α and PDK1 (in ccRCC tissues and normal renal tissues). Nonparametric Student’s t-test was used for two-group comparison (invasion, migratory, and animal model). Analysis of variance of repeated measures was used to evaluate the growth curves of the RTCA results. A P-value of <0.05 was considered significant.

## Results

### Western Blot Analysis of HIF-1α Expression in Human ccRCC Tissues

**Figure 1:**
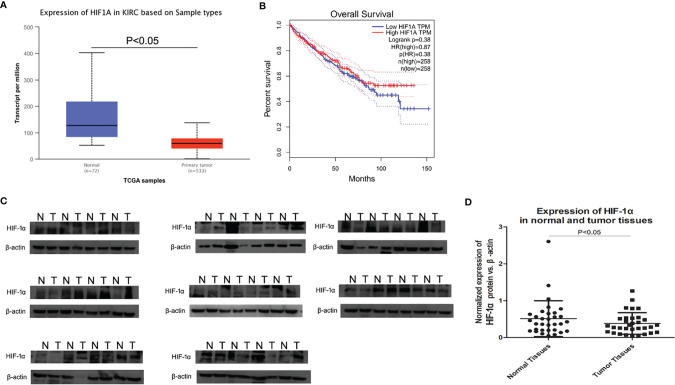
Expression of HIF-1α by TCGA and western blot in ccRCC tissues and normal renal tissues. **(A)** HIF-1α expression was significantly down‐regulated in ccRCC tissue compared to that in non‐tumor tissues, P <0.001. **(B)** HIF-1α expression had no correlation with overall survival in ccRCC. **(C)** The average HIF-1α expression for all studied ccRCC tissues and corresponding normal rnal tissues (32 cases) by western blot. **(D)** Scatter plot (vertical) graphs describe conspicuous HIF-1α downregulation in ccRCC tissues in comparison with normal tissues (P <0.05). β-actin was used as the control for normalization. “N” represented normal renal tissues; “T” represented ccRCC tissues.

As shown in **Figure 1A**, TGCA database presented that HIF-1α expression was significantly down‐regulated in ccRCC tissues compared to that in non‐tumor tissues. Furthermore, as shown by the Kaplan–Meier curve, there was no significant correlation between HIF-1α mRNA expression and overall survival status (**Figure 1B**). According to the analysis in The Human Protein Atlas database, the expression of HIF-1α is almost weak to moderate (25–75%) in normal renal tissues. However, in ccRCC tissues HIF-1α shows a weak expression of <25% and a certain number of ccRCC tissues show a very weak expression of HIF-1α (at the link of https://www.proteinatlas.org/). Then western blot assay was used to evaluate the expression of HIF-1α in 32 cases of human ccRCC (the clinical tissue specimens were all collected from The First Affiliated Hospital of China Medical University). All results of western blot are showed in **Figure 1C**. HIF-1α protein was overwhelmingly expressed in the normal renal tissues while it was expressed at lower levels in the ccRCC areas (among the 32 cases, 21 cases showed higher expression in normal renal tissues, P <0.05, **Figure 1D**).

### HIF-1α and PDK1 Expression and Location in Renal Cell Lines

**Figure 2:**
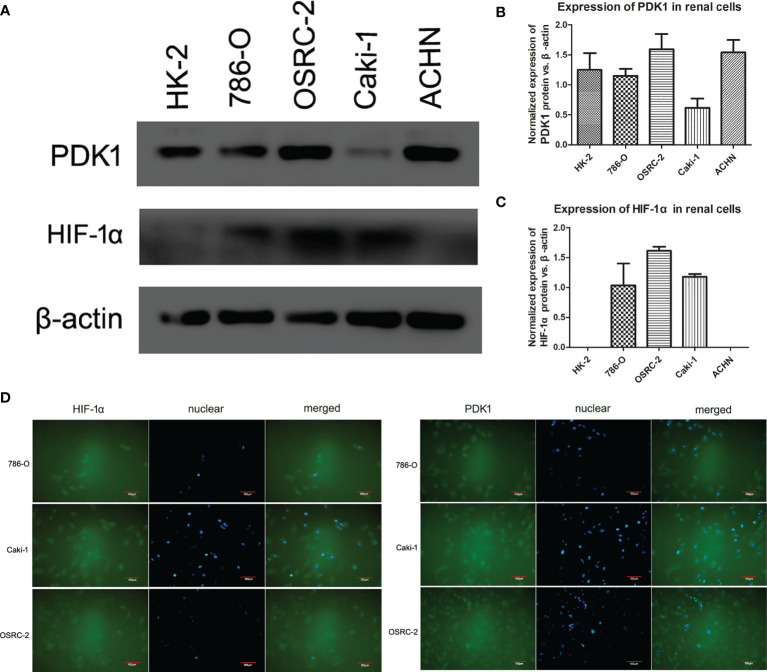
Expression and location of HIF-1α and PDK1 in renal cell lines. **(A–C)** HIF-1α and PDK1 expression at the protein level in various cell lines (HK-2, 786-O, OSRC-2, Caki-1, and ACHN) was confirmed by western blot. β-actin was regarded as the reference. No expression of HIF-1α was observed in HK-2 and ACHN cells. PDK1 expression was sharply higher in ACHN and OSRC-2 cells than in HK-2, 786-O, or Caki-1 cells. **(D)** Using immunofluorescence analysis, the expression of HIF-1α and PDK1 was located in both the cytoplasm and cell nucleus (green color). The locations were almost the same in 786-O, Caki-1, and OSRC-2 cells.

To investigate HIF-1α and PDK1 expression in renal cancer cells, a western blot assay was employed (**Figure 2A**). Western blot assays revealed the expression of PDK1 at different levels in various renal cancer cell lines; among the cells, PDK1 protein expression was found at a significantly higher level in ACHN and OSRC-2 cells (**Figure 2B**, P <0.05). Meanwhile, with antibodies specific to HIF-1α, the HIF-1α protein demonstrated almost no expression in HK2 and ACHN cells, and OSRC-2 cells showed the highest expression level (**Figure 2C**, P <0.05). Immunofluorescence was used to examine HIF-1α and PDK1 expression and localization. Immunofluorescence results showed that HIF-1α was mainly expressed in both the cytoplasm and nucleus in 786-O, Caki-1, and OSRC-2 cell lines, and PDK1 also showed similar results (**Figure 2D**).

### Decreasing Proliferation, Migration and Invasion Ability After Transfection With HIF-1α siRNA

**Figure 3:**
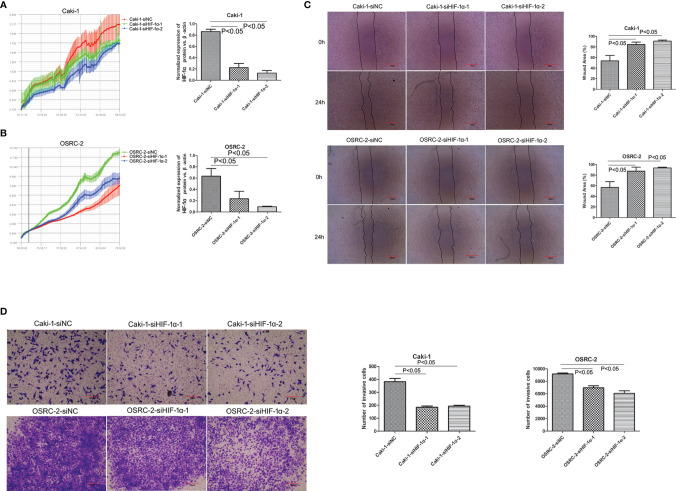
Knockdown of HIF-1α attenuated the effect on cellular proliferations, migrations and invasion (both Caki-1 and OSRC-2 cells). Downregulation of HIF-1α inhibited cell proliferation as shown by the RTCA results (P <0.05). **(A)** Red line: Caki-1-siNC, green line: Caki-1-siHIF-1α-1, blue line: Caki-1-siHIF-1α-2; **(B)** Green line: OSRC-2-siNC, red line: OSRC-2-siHIF-1α-1, blue line: OSRC-2-siHIF-1α-2. **(C)** Comparison of scratch width to verify the migratory capability of HIF-1α-siRNA-transfected cells and NC-siRNA-transfected cells. Knockdown of HIF-1α sharply inhibited the migration of both Caki-1 and OSRC-2 cells (P <0.05). **(D)** Comparison of penetrability in Transwell chambers to verify the invasive capability of HIF-1α-siRNA-transfected cells and NC-siRNA-transfected cells. Knockdown of HIF-1α sharply suppressed the invasion of both Caki-1 and OSRC-2 cells (P <0.05).

To assess the effects of HIF-1α silencing on the proliferation, migration, and invasion of Caki-1 and OSRC-2 cells, an RTCA assay, wound healing assay, and Transwell assay were used to determine the changes in the biological characteristics of Caki-1 and OSRC-2 cells. Knockdown of HIF-1α expression was confirmed by western blot (**Figure 6B**). HIF-1α-siRNA induced a significant decrease in the proliferation of both Caki-1 and OSRC-2 cells at more than 68 h compared to the negative control (NC-siRNA) on the basis of RTCA results (**Figures 3A, B**, P <0.05). The outcomes of the wound healing assay suggested that the cells migrated slowly to close in the Caki-1 or OSRC-2 (HIF-1α-siRNA) group compared with the Caki-1 or OSRC-2 (NC-siRNA) group (**Figure 3C**, P <0.05). Moreover, a Transwell (chambers with Matrigel) invasion assay was performed to examine the effects of HIF-1α silencing on the invasive abilities of Caki-1 and OSRC-2 cells. The results indicated that the ability of Caki-1 and OSRC-2 cells to pass through the Transwell chambers was significantly decreased in the Caki-1 or OSRC-2 (HIF-1α-siRNA) group compared with the Caki-1 or OSRC-2 (NC-siRNA) group (**Figure 3D**, P <0.05). In general, the above results suggested that HIF-1α silencing might suppress the proliferative, migratory and invasive abilities of renal cancer cells; that is, HIF-1α might participate in the progression of renal cancer.

### Growth of Tumors Derived From OSRC-2-sh-HIF-1α Cells Compared With OSRC-2-shNC Cells

**Figure 4:**
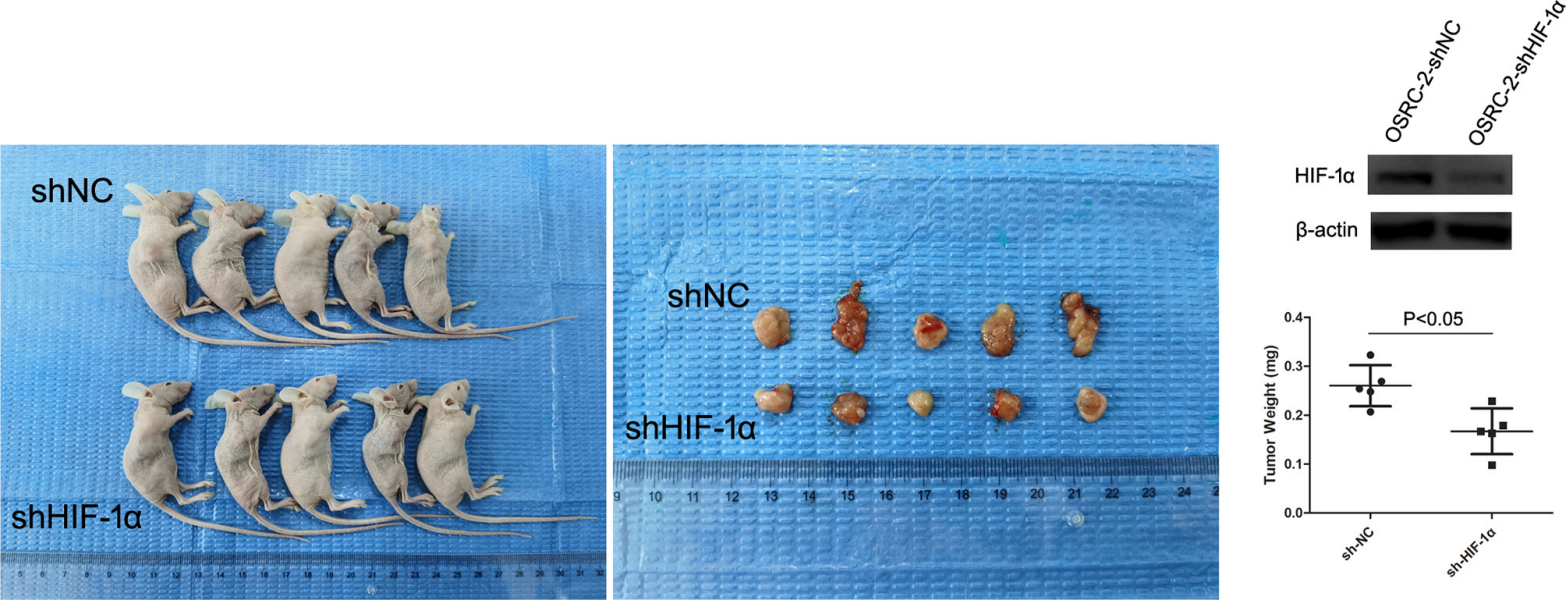
Nude mouse experiment. The expression of HIF-1α was significantly downregulated in OSRC-2 cells transfected with HIF-1α-shRNA compared to NC-shRNA-transfected cells (P <0.01). The representative graphs show that tumors formed. Knockdown of HIF-1α suppressed tumor volumes (P = 0.0159).

To confirm further that HIF-1α might serve as a tumor promoter in renal cancer, we examined its function in a xenograft tumor model (nude mice). First, HIF-1α-shRNA knockdown efficiency was detected by western blot (**Figure 4**). Then, we found that stable transfection of HIF-1α-shRNA into OSRC-2 cells led to markedly decreased growth and tumor weight of xenograft tumors compared with the NC-shRNA group. Tumor weights were as follows: HIF-1α-shRNA group: 0.167 ± 0.047 g, NC-shRNA group: 0.260 ± 0.042 g, P = 0.0159 (**Figure 4**). In addition, our group has been committed to the research of hZIP1. According to our previous study, hZIP1 overexpression decreased the formation of tumors (ccRCC cells) in nude mice by targeting GAS5/miR-223 (18). In one study, the researcher declared that HIF-1α could be inhibited by zinc to improve the therapeutic effect of tumor (19). So we conducted the ccRCC cells with hZIP1 overexpression for the follow-up study.

### Identification of Stable Clones of hZIP1 and Expression of HIF-1α After hZIP1’s Overexpression

**Figure 5:**
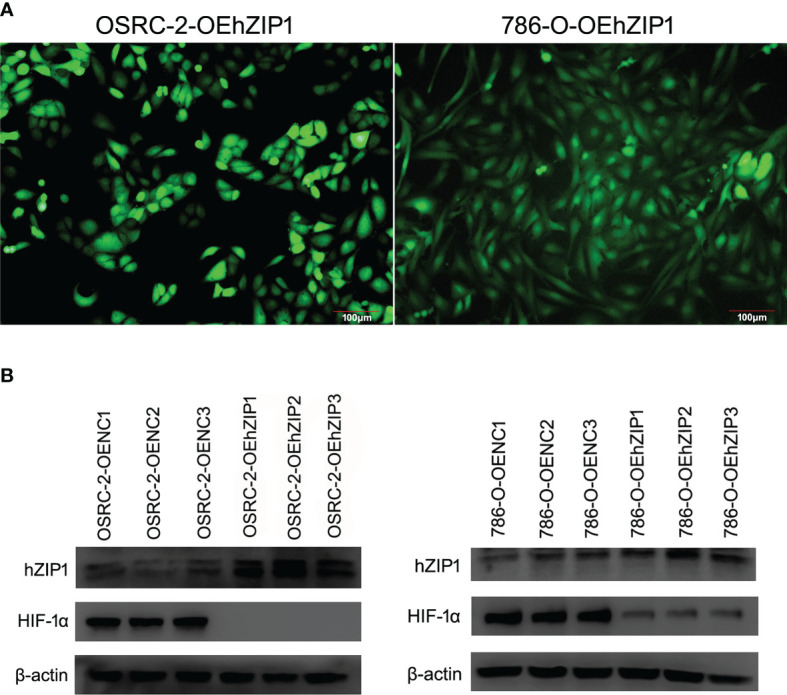
Overexpression of hZIP1 inhibited the expression of HIF-1α. **(A)** Fourteen days after the lentiviral transfection, the OSRC-2/786-O cells were successfully overexpressed with hZIP1 under a fluorescent inverted microscope. **(B)** We stably transfected the OSRC-2 cells/786-O cells with hZIP1 lentivirus, and changes in HIF-1α protein levels were obtained by western blot analysis and compared with those in cells stably transfected with negative control lentivirus (P <0.05).

As shown in **Figure 5A**, the positive cells transfected successfully of hZIP1 expression plasmid (lentivirus) in OSRC-2 and 786-O cells was evaluated by fluorescence microscopy 14 days after transfection and then selection with 2 μg/ml puromycin. The expression of hZIP1 in OSRC-2-OEhZIP1 cells and OSRC-2-OENC cells was confirmed by western blot assays, along with 786-O cells. As shown in **Figure 5B**, and western blot analysis using β-actin as a loading control revealed that hZIP1 protein levels substantially increased in OSRC-2-OEhZIP1 cells (786-O-OEhZIP1 cells) compared with OSRC-2-OENC cells (786-O-OENC cells). However, it surprised us that HIF-1α presented no expression after hZIP1 overexpression in OSRC-2 cells. Meanwhile in the 786-O-OEhZIP1 cells, HIF-1α showed a sharp decrease (**Figure 5B**). In the supplementary experiment, RT-PCR analysis demonstrated that HIF-1α mRNA levels had not changed after hZIP1 overexpression in OSRC-2 and 786-O cells (**Figure S2**).

### Change of hZIP1 and HIF-1α Affected Glycolysis and Tumorous Features in Renal Cancer Cells

**Figure 6:**
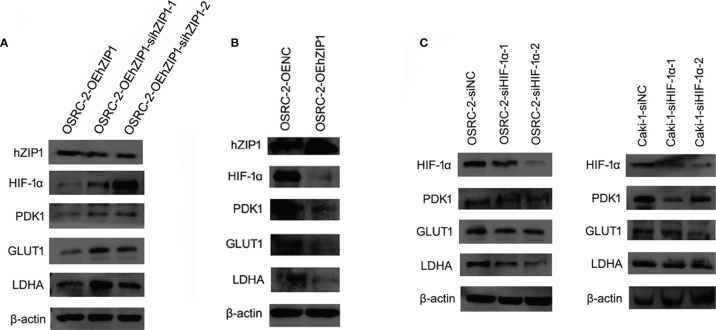
Effect of hZIP1-siRNA and HIF-1α-siRNA on renal cancer cells. **(A)** First, the siRNA knockdown efficiency on the OSRC-2-OEhZIP1 cells was analyzed by western blot (P <0.01). Western blot results also indicated that HIF-1α, PDK1, GLUT1 and LDHA expression significantly declined after loss of hZIP1 (P <0.05). **(B)** Second, hZIP expressing plasmid was selected to increase hZIP1 expression. PDK1, GLUT1, and LDHA protein levels were decreased upon hZIP1 overexpression (P <0.05). **(C)** Third, HIF-1α-siRNA was selected to downregulate HIF-1α expression. PDK1, GLUT1, and LDHA protein levels were decreased upon HIF-1α downregulation (P <0.05).

The hZIP1 protein acted as a tumor suppressor in ccRCC according to our previous work (17). We believe that after the knockdown of hZIP1, the malignant progression of renal cancer could be expedited. To test our prediction, we used Lipo3000 to transfect siRNA to knockdown ZIP1 in OSRC-2-OEhZIP1 cells. We had tried to knockdown hZIP1 in control cells, but the results were far from the results in hZIP1 overexpression cells (**Figure S3**). So hZIP1 knockdown in hZIP1 overexpression cells could present larger differences of hZIP1. Therefore, the change of downstream genes was more significant. Western blot was performed to confirm the knockdown efficiency, and then the expression of HIF-1α increased after hZIP1 knockdown. The glycolysis-related proteins, PDK1, GLUT1, and LDHA, also showed an increasing trend (**Figure 6A**). hZIP1 overexpression decreased HIF-1α expression according to **Figure 5B**, and PDK1, GLUT1, and LDHA showed an decreasing trend (**Figure 6B**). Additionally, another siRNA was chosen to knockdown HIF-1α, and much to our surprise and delight, it was accompanied by the downregulation of HIF-1α and the expression of PDK1 was decreased. GLUT1 and LDHA expression exhibited a declining trend (**Figure 6C**). These results suggested that hZIP1 and HIF-1α had major impacts on the tumorous process by influencing glycolysis in renal cancer cells.

### hZIP1 Inhibited the Entry of NF-kB1 Into Cell Nucleus

**Figure 7:**
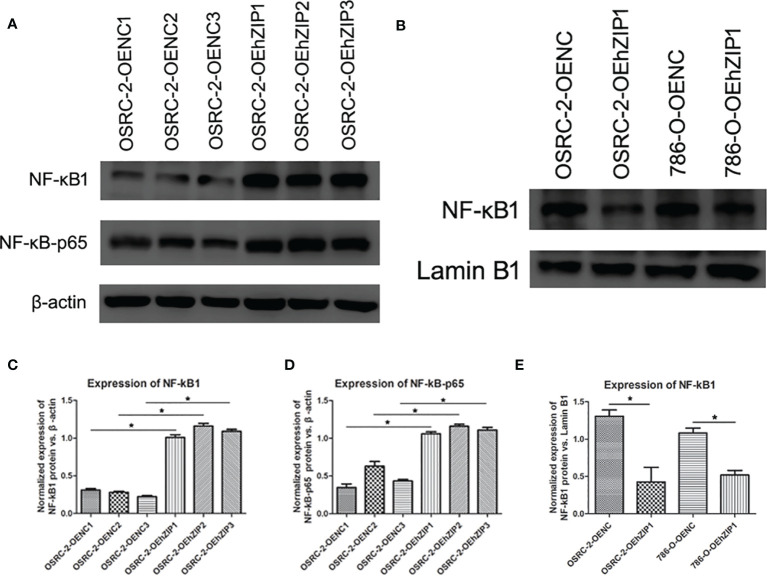
hZIP1affected NF-kB pathway. **(A, C, D)** Western blot results demonstrated that NF-kB1 and NF-kB-p65 expression significantly increased after expression of hZIP1 (“*” represented P <0.05). **(B, E)** Additionally, less NF-kB1 moved into the cell nucleus was detected in OSRC-2-OEhZIP1 cells and 786-O-OEhZIP1 cells (“*” represented P <0.05).

To further evaluate the effects of decreased HIF-1α expression through hZIP1 overexpression, we examined NF-*k*B pathway-associated proteins. The results were basically satisfying that both NF-*k*B1 and NF-*k*B-p65 presented an upward trend (**Figures 7A, C, D**). Nucleoproteins were also extracted, and then we observed a decreased trend of NF-*k*B1 that moved into the cell nucleus in OSRC-2-OEhZIP1 cells and 786-O-OEhZIP1 cells (**Figures 7B, E**). These results indicated that hZIP1 inhibited ccRCC progression by suppressing NF-*k*B pathway.

## Discussion

The most common adult renal cancer is ccRCC (20). The fundamental pathogenic mechanism includes both external and internal risks, such as smoking, obesity, and oxidative stress (21, 22). There are reports that the hypoxia pathway plays a crucial role in the development of many cancers including renal clear cell cancer, which has been stated to be VHL gene mutate (10). The best-known hypoxia-inducible factor, 1α (HIF-1α), has been confirmed to play important roles in transcription initiation during the tumor progression (23). First, in our study, we examined the expression of HIF-1α in both ccRCC cells and tissues. We found that HIF-1α was downregulated in ccRCC tissues, it was consistent with the findings in TCGA platform and The Human Protein Atlas database, but there was no significant correlation between HIF-1α expression and overall survival status. Then we found that OSRC-2 and Caki-1 cells expressed high levels of HIF-1α while no expression was observed in ACHN and HK-2 cells. Immunofluorescence results showed that HIF-1α and PDK1 were located in both cytoplasm and nucleus. In regard to the western blot results, we demonstrated a lower expression of HIF-1α in ccRCC tissues than in the corresponding normal tissues. This result suggested that HIF-1α tended to play different roles in normal renal or cancerous renal, it also played a role in physiological processes of normal renal and further verification is needed.

Second, referring to our previous results, hZIP1 plays a vital role in the progression of renal cancer, knockdown of hZIP1 increases the proliferation and invasion of ACHN cells, and overexpression of hZIP1 diminishes the formation of tumors in nude mice by targeting GAS5/miR-223 (17, 18). Moreover, zinc induced HIF-1α proteasomal degradation and suppressed VEGF expression in prostate cancer and glioblastoma (24). After hZIP1 silencing by hZIP1-siRNA, HIF-1α augmented, and PDK1, GLUT1, and LDHA also showed an increasing tendency (cell experiments were performed using Caki-1 and OSRC-2). PDK1 plays a significant role in aerobic glycolysis and regulates the biological behavior of tumor cells (25–27). According to the analysis of TCGA database, HIF-1α was significantly down‐regulated in ccRCC tissues compared to that in normal renal tissues. As for PDK1, its expression was significantly up‐regulated in ccRCC tissues compared to that in normal renal tissues. But as shown by the Kaplan–Meier curve, patients with low PDK1 mRNA expression exhibited a lower overall survival compared to patients with high PDK1 mRNA expression (**Figure S4**). HIF-1α was not exactly negative related to PDK1, and needs further exploration. When we knocked down the expression of HIF-1α with HIF-1α-siRNA, the proliferative, migratory and invasive abilities were inhibited, and the same was true of the expression of GLUT1 and LDHA. The above results confirmed that HIF-1α was involved in the development of renal cancer and played a significant role in glycolysis in renal malignancy. Similarly, the nude mouse experiment substantiated that HIF-1α absence contributed to reduced tumor formation, which conformed to the results mentioned above. HIF-1α is reported to suppress apoptosis and to promote tumorous progression in renal cancer cells (28, 29); especially associated with metastatic tumors of ccRCC (30). Moreover, the role of HIF-1α in the pathological process is related to cellular energy metabolism (31). The above results were consistent with our outcomes. However, some studies held opposing views; they considered HIF-1α to act as a tumor suppressor to inhibit cancerous behavior in renal cancer (24, 32, 33). Our study suggested that HIF-1α presented a lower expression in ccRCC tissues compared with normal renal tissues, but HIF-1α presented almost no expression in immortalized renal tubular epithelial cell HK-2, and these seemed contradictory. We suspected HIF-1α might be involved in renal physiological behaviors and regulated oncogenesis. As for the reason why the function of HIF-1α in ccRCC was identified as an oncogene or a tumor suppressor, we speculated that HIF-1α might play different roles at the beginning or advanced stage of ccRCC. The role and mechanism of HIF-1α involved in ccRCC progression was complicated, that needed further exploration.

Third, our previous work confirmed that overexpression of hZIP1 inhibited proliferation, cell cycle progression, and invasion and induced apoptosis of renal cancer cells (18). At this stage of our experiment, after hZIP1 overexpression, HIF-1α decreased. But the HIF-1α mRNA level made no change after hZIP1 overexpression. That suggested this regulation is mainly at the post transcriptional level. Nardinocch et al. confirmed that zinc is used as an inhibitor of HIF-1α in human tumors to activate VEGF, MDR1 to enhance the effect of anticancer therapies (19). This was consistent with our results. Our mechanism studies showed that hZIP1 inhibited tumor processes by upregulating NF-*k*B1 and NF-*k*B-p65, which was highly likely for the reason of changes in Zn^2+^ levels.

Furthermore, Ahmmed et al. suggested that NF-*k*B is downregulated in lung cancer, with the HIF-1α upregulation caused by PTX3 being NF-*k*B-dependent (34). Moreover, the tumor microenvironment of pancreatic cancer has been confirmed to be highly hypoxic. Hypoxia not only results in the accumulation of HIF-1α, but also activates the NF-*k*B transcription factors, PKM2 promotes tumor angiogenesis by regulating HIF-1α through NF-*k*B activation (35), HIF-1α activates NF-*k*B, NF-*k*B controls HIF-1α transcription and that HIF-1α activation may be concurrent with inhibition of NF-*k*B (36). Additionally, our results showed that the NF-*k*B1 protein travelled to the cell nucleus declined after hZIP1 overexpression. One of the roles of NF-kB is to enter the cell nucleus and promote tumor progression (37). Maybe hZIP1 overexpression inhibited NF-*k*B signaling and then suppressed HIF-1α transcription in ccRCC.

Taken together, HIF-1α was involved in ccRCC progression and related to anaerobic glycolysis. The inhibition of renal cancer induced by hZIP1 might be connected with HIF-1α (Zn^2+^ might act as an intermediary) and even metabolic changes, which requires further investigation. We hypothesized that hZIP1 inhibited renal tumorous progression by influencing NF-*k*B pathway and HIF-1α-induced metabolic reprogramming. However, our present study had a few boundedness. The exploration of the mechanism was essential to sustain our findings, where the next step was confirming the exact mechanism. According to our results and references, we speculated the interaction of hZIP1 and HIF-1α might be related to metabolism and several classical pathways (such as PI3K‐Akt- mTOR, Wnt, and NF‐kB pathways).Then metabolomic analysis and transcriptome analysis will be conducted to enrich our findings. ccRCC patients at an early stage has better prognosis, but a significant portion of the patients were diagnosed with giant ccRCC or long-distance metastasis. Surgical debulking, targeted biological drugs or interventional therapy may be appropriate for them. Nevertheless, uncertainties and research questions remain (38). In recent years, immune checkpoint inhibitors therapy (PD-L1) and targeted therapy (kinase inhibitors) have been introduced into the treatment of metastatic renal cancer, and produced good effects (39, 40). However, drug resistance is always a problem. Our study on the mechanism of hZIP1 can suggest new biomarkers and targets on ccRCC therapies. Meanwhile, it can provide a theoretical basis for the study of renal cancer development.

### Conclusion

Our results suggest that hZIP1 overexpression inhibits ccRCC process by suppressing NF‐kB/HIF-1α pathway.

## Data Availability Statement

Publicly available datasets were analyzed in this study. This data can be found here: http://gepia.cancer-pku.cn/detail.php.

## Ethics Statement

The studies involving human participants were reviewed and approved by the Ethics Committee of China Medical University. The patients/participants provided their written informed consent to participate in this study. The animal study was reviewed and approved by the Ethics Committee of China Medical University.

## Author Contributions

The study was conceived by XD and BZ. Experiments were come into effect by XD, BZ, ZG, YY, and BL. Statistical analysis was carried out by BZ. Manuscript was written by XD and BZ. All authors contributed to the article and approved the submitted version.

## Funding

This work was supported by the National Natural Science Foundation of China (81902591).

## Conflict of Interest

The authors declare that the research was conducted in the absence of any commercial or financial relationships that could be construed as a potential conflict of interest.

## Publisher’s Note

All claims expressed in this article are solely those of the authors and do not necessarily represent those of their affiliated organizations, or those of the publisher, the editors and the reviewers. Any product that may be evaluated in this article, or claim that may be made by its manufacturer, is not guaranteed or endorsed by the publisher.

## Abbreviations

ccRCC, clear cell renal cell carcinoma; RTCA, real-time cell analysis; HIF, hypoxia-inducible factor; VEGF, vascular endothelial cell growth factor; FBS, fetal bovine serum; RIPA, radioimmunoprecipitation assay; PMSF, phenylmethanesulfonyl fluoride; PVDF, polyvinylidene fluoride.
