# Supplementary figures and images for: hZIP1 Inhibits Progression of Clear Cell Renal Cell Carcinoma by Suppressing NF-kB/HIF-1α Pathway
Source: Front Oncol. 2021 Dec 2;11:759818. doi: 10.3389/fonc.2021.759818 (PMC8674186; doi:10.3389/fonc.2021.759818)

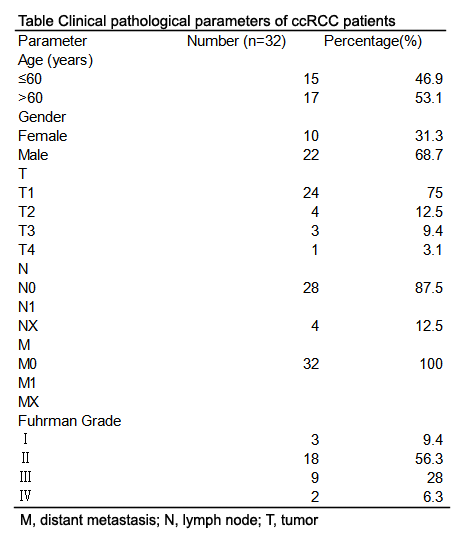

Supplement: Supplementary file 1 [file Image_1.tif]

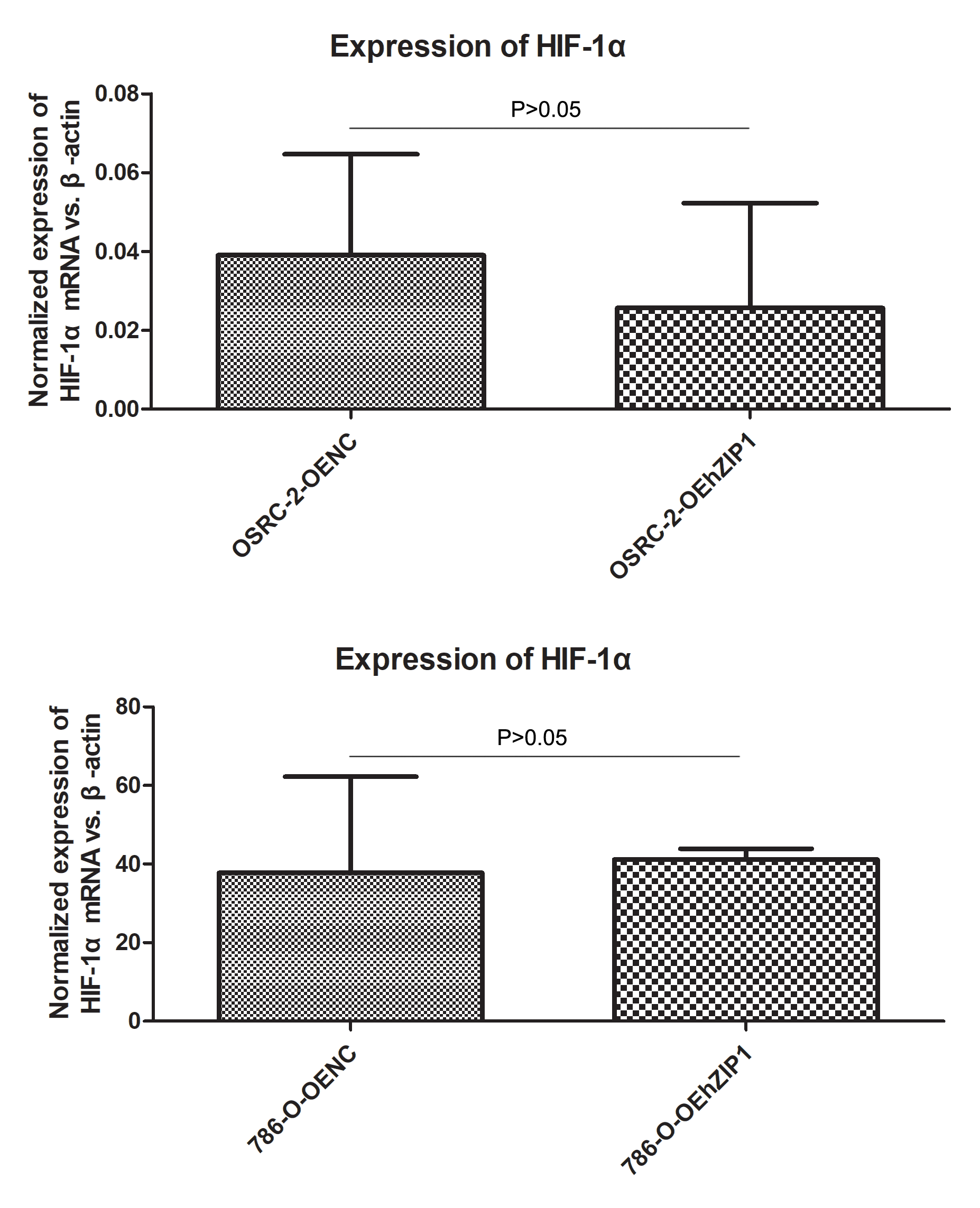

Supplement: Supplementary file 2 [file Image_2.tif]

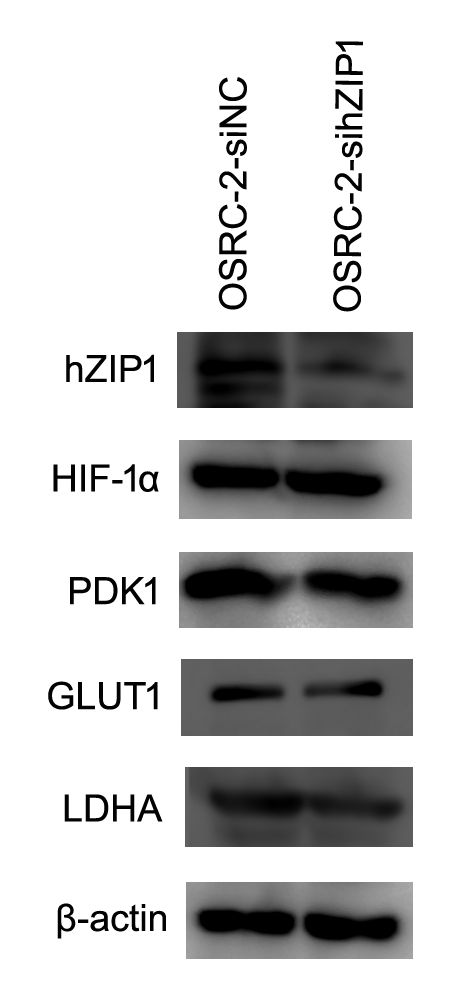

Supplement: Supplementary file 3 [file Image_3.tif]

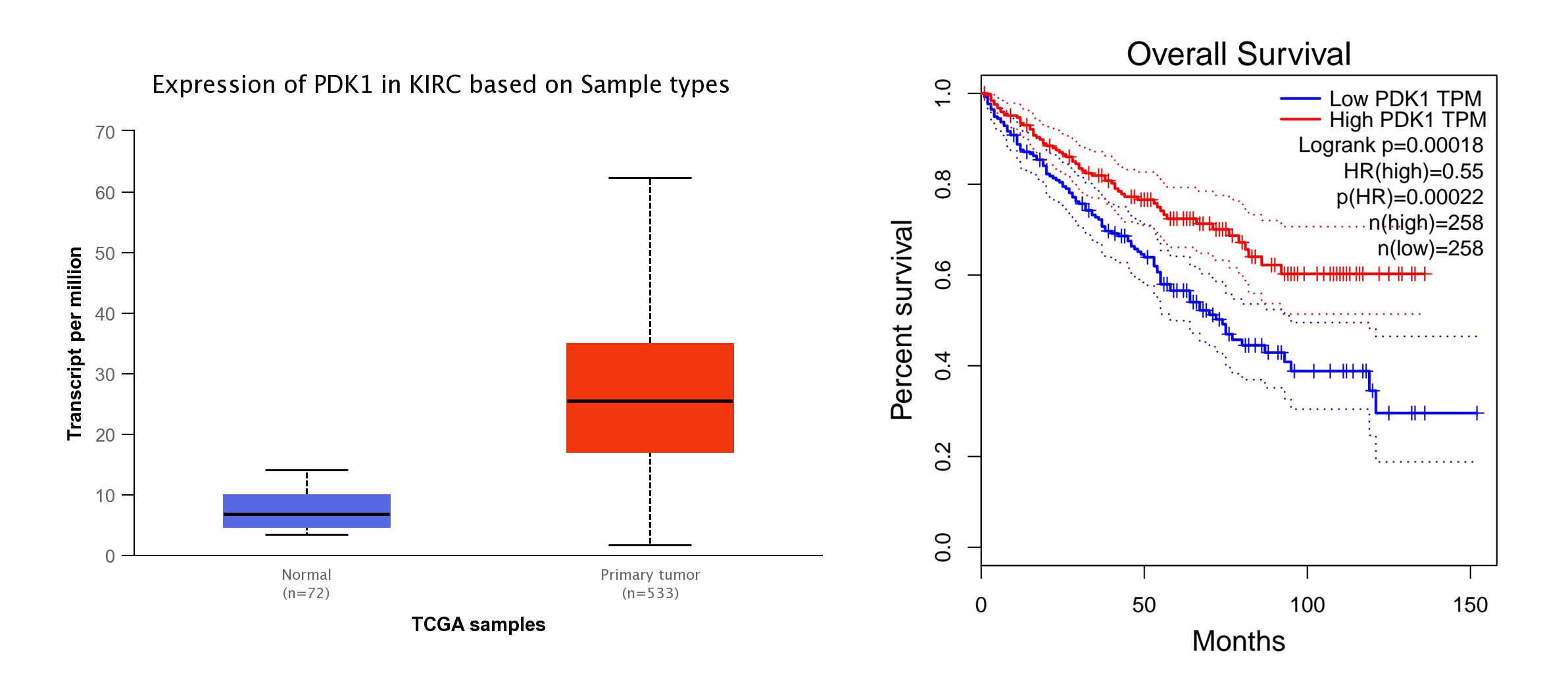

Supplement: Supplementary file 4 [file Image_4.tif]
